# Safety and feasibility of trans‐venous cardiac device extraction using conscious sedation alone—Implications for the post‐COVID‐19 era

**DOI:** 10.1002/joa3.12637

**Published:** 2021-09-22

**Authors:** Thomas Lachlan, Hejie He, Hesham Aggour, Preet Sahota, Samuel Harvey, Kiran Patel, Will Foster, Shamil Yusuf, Sandeep Panikker, Tarv Dhanjal, Uday Dandekar, Thomas Barker, Jitendra Parmar, Michael Kuehl, Faizel Osman

**Affiliations:** ^1^ Department of Cardiology University Hospitals Coventry & Warwickshire NHS Trust Coventry UK; ^2^ University of Warwick (Medical School) Coventry UK; ^3^ Worcester Royal Hospital Worcester UK

**Keywords:** cardiac implantable electronic devices, conscious‐sedation, Fentanyl, lead extraction, Midazolam

## Abstract

**Background:**

Transvenous lead extraction (TLE) for implantable cardiac‐devices is traditionally performed under general anesthesia (GA). This can lead to greater risk of exposure to COVID‐19, longer recovery‐times and increased procedural‐costs. We report the feasibility/safety of TLE using conscious‐sedation alone with immediate GA/cardiac‐surgery back‐up if needed.

**Methods:**

Retrospective case‐series of consecutive TLEs performed using conscious‐sedation alone between March 2016 and December 2019. All were performed in the electrophysiology‐laboratory using intravenous Fentanyl, Midazolam/Diazepam with a stepwise approach using locking‐stylets/cutting‐sheaths, including mechanical‐sheaths. Baseline patient‐characteristics, procedural‐details and TLE outcomes (including procedure‐related complications/death) were recorded.

**Results:**

A total of 130 leads were targeted in 54 patients, mean age ± SD 74.6 ± 11.8years, 47(87%) males; dual‐chamber pacemakers (n = 26; 48%), cardiac resynchronization therapy‐defibrillators (n = 17; 31%) and defibrillators (n = 8; 15%) were commonest extracted devices. Mean ± SD/median (range) lead‐dwell times were 11.0 ± 8.8/8.3 (0.3‐37) years, respectively. Extraction indications included systemic infection (n = 23; 43%) and lead/pulse‐generator erosion (n = 27; 50%); mean 2.1 ± 2.0 leads were removed per procedure/mean procedure‐time was 100 ± 54 min. Local anesthetic (LA) was used for all (mean‐dose: 33 ± 8 ml 1% lidocaine), IV drug‐doses used (mean ± SD) were: midazolam: 3.95 ± 2.44 mg, diazepam: 4.69 ± 0.89 mg and fentanyl: 57 ± 40 µg. Complete lead‐extraction was achieved in 110 (85%) leads, partial lead‐extraction (<4 cm‐fragment remaining) in 5 (4%) leads. Sedation‐related hypotension requiring IV fluids occurred in 2 (managed without adverse‐consequences) and hypoxia requiring additional airway‐management in none. No procedural deaths occurred, one patient required emergency cardiac surgery for localized ventricular perforation, nine had minor complications (transient hypotension/bradycardia/pericardial effusion not requiring intervention).

**Conclusion:**

TLE undertaken using LA/conscious‐sedation was safe/feasible in our series and associated with good clinical outcome/low procedural complications. Reduced risk of aerosolization of COVID‐19 and quicker patient recovery/reduced anesthetic risk are potential benefits that warrant further study.

## Graphical abstract

TLE undertaken using LA/conscious‐sedation was safe/feasible in our series and associated with good clinical outcome/low procedural complications. Reduced risk of aerosolisation of Covid‐19 and quicker patient recovery/reduced anesthetic risk are potential benefits that warrant further study.

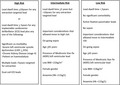

## 1 INTRODUCTION

Use of cardiac implantable electronic devices (CIEDs) has been steadily expanding worldwide given these devices improve morbidity and mortality.^1^, ^2^ However, increasing implant rates have led to an increase in CIED related complications and consequently increased need for device extractions. Presently, ~30,000 cardiac device extractions are performed worldwide, with infection a leading cause followed by lead malfunction. Infection‐related extractions have risen 30%‐50% between 2006 and 2012.^3^ Extraction techniques have evolved with trans‐venous lead extraction (TLE) becoming the treatment of choice. TLE remains a challenging procedure with high procedural risks related to cardiac tamponade, vascular bleeding^4^, ^5^ and peri‐operative mortality correlated with device infection.^6^, ^7^, ^8^ The 2017 Heart Rhythm Society (HRS) TLE consensus guidelines strongly advocate the importance of a collaborative and multidisciplinary approach to address lead extraction management optimizing both safety and efficacy.^9^ The logistic approach to TLE differs across different hospitals. Cases are often performed under general anesthesia (GA), but this carries its own risks to patients, who are typically at high risk. In addition, in the current pandemic there is increased risk of COVID‐19 infection transmission from aerosolization associated with GA.^10^ There are very limited data on the use of conscious sedation alone for such procedures. We have developed a protocol for performing TLE using conscious‐sedation by default and evaluated the feasibility and safety of this approach in our study.

## 2 METHODS

### 2.1 Patient population

We started our policy of performing TLE using conscious‐sedation alone at University Hospitals Coventry and Warwickshire NHS Trust, a large tertiary hospital in the UK, from March 2016. We attempted TLE using conscious‐sedation alone in all consecutive patients as the primary approach irrespective of lead type, lead dwell‐time or co‐morbidities unless a patient was known to need concomitant cardiac surgery (e.g., heart valve surgery/bypass grafting) from the outset. Rapid response back up from Anaesthesiology and Cardiothoracic Surgery was always available with facilities to convert the case to GA if needed, as well as facilities to open the chest in the electrophysiology (EP) cath‐lab if required.

We performed a retrospective study of all TLE cases that were planned to be performed using conscious sedation alone from the outset between March 2016 and December 2019. Patients with recently implanted leads (less than 6 months), requiring lead explant by traction alone with no extraction tools, were excluded. Lead extraction procedures were defined in accordance with the HRS TLE guideline^9^ and European Heart Rhythm Association (EHRA) consensus statement.^11^ Complete procedural success was defined as removal of all lead material from the vascular space with no permanent/disabling complications. Clinical procedural success was defined as removal of all targeted leads with retention of ≤4 cm of lead material that did not cause undesired outcomes. Procedural failure was defined as inability to reach complete procedure or clinical procedural success irrespective of clinical outcome alone. Major/minor complications were defined as per guidelines.^9^, ^11^ Demographic, clinical, and procedural data were collected on all and written informed consent obtained from all. The study was approved by our institution’s research committee and was in accordance with the Declaration of Helsinki. Patients were involved by being aware they were undergoing a high risk procedure and being invited to participate in research which is on‐going on optimal management strategies at our center. There was no direct patient involvement in outcome measures or study design but all patients provided informed consented pre‐procedure.

### 2.2 Pre‐procedural assessment

**FIGURE 1:**
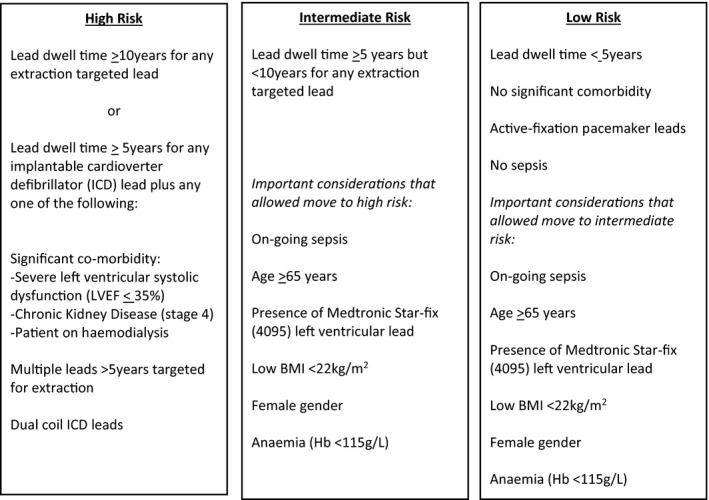
Pre‐extraction risk stratification protocol

Prior to the extraction procedure all patients were evaluated in the device clinic if an elective extraction or if an in‐patient, by a consultant Electrophysiologist. Evaluation included a comprehensive clinical and device history, pre‐procedural risk stratification, determination of need for concomitant cardiothoracic surgery, and assessment of device re‐implantation. Patients were discussed in a multi‐disciplinary meeting before extraction with both cardiologist and cardiac surgeon present. We classified patient risk pre‐procedure into low, medium or high risk according to baseline features (Figure 1); these were determined from previous studies and guidelines.^9^, ^11^ The factors that were used in our classification included lead dwell time, number and type of leads, gender, age of patient at time of extraction, comorbidity (such as severe left ventricular systolic dysfunction, chronic kidney disease/on hemodialysis), on‐going active sepsis, low body mass index (defined <22 kg/m^2^), and anemia (defined as hemoglobin concentration <115 g/L). Those patients with any lead implanted ≥10‐year or with an implantable cardioverter defibrillator (ICD) lead ≥5 years were deemed high risk. Those with lead dwell times between 5 and 10 years were deemed intermediate risk, but operators were allowed to upgrade to high risk if a combination of features were present at their discretion; these included significant co‐morbidity, multiple targeted leads, dual coil ICD leads. Those with leads implanted <2 years without any high or intermediate risk features were deemed low risk (Figure 1).

For patients in the intermediate and low risk groups the TLE was performed with a cardiac surgeon and anesthetist on site at the hospital and available if needed in an emergency, but not present in the EP‐lab. Those in the high risk group were performed with the cardiac surgeon and anesthetist on standby close to the EP‐lab with facilities to immediately convert to GA and open the chest/commence cardiac‐bypass if required. If needed both transthoracic echocardiography (TTE) and trans‐esophageal echocardiography (TOE) were immediately available. Both the cardiac surgeon and anesthetist were able to perform other non‐operating duties during this time (such as administration tasks) ensuring their time was utilized productively if they were not needed during the case.

### 2.3 Patient monitoring and sedation

All procedures were performed in our EP laboratory by two experienced Cardiologists trained in TLE. Two fully trained experienced cath‐lab nurses assisted, one monitoring patient vital signs and administering intravenous (IV) drugs with supervision from the cardiologists; all nurses had full advanced cardiac life support training. Patients were prepared with sterile full chest and femoral access preparation and monitored with continuous electrocardiogram (ECG), invasive blood pressure monitoring (via radial or femoral [4F] artery access), oxygen saturation/carbon dioxide levels and, if needed, arterial blood gas analysis. All received peripheral vein cannulas and one/two femoral venous sheaths pre‐extraction. Level of sedation was classified according to American Society of Anaesthesiology guidelines and defined as drug‐induced depression of consciousness during which patients respond purposefully to verbal commands (either alone or accompanied by light tactile stimulation) with no interventions required to maintain a patent airway and adequate spontaneous ventilation with maintained cardiovascular function.^12^

All patients received IV paracetamol 1 g pre‐op and most had analgesia using IV fentanyl (25 µg boluses, max 20 µg), IV diazepam (2.5 mg boluses, max 5 mg), and/or IV midazolam (1 mg boluses, max 10 mg) administered as required. Deep sedation (defined as being unresponsive to vocal stimuli, tolerating an oropharyngeal airway but breathing spontaneously) was avoided. Fentanyl was used for analgesia and not to aid lead extraction by veno‐dilation. Doses were adjusted as indicated by patients’ comfort level and hemodynamic status. If necessary, patients received IV saline infusion to achieve and maintain systolic blood pressure above 90 mm Hg. Oxygen was applied via an oxygen mask and flow adjusted to achieve oxygen saturation levels >90%. If peripheral oxygen saturation decreased <90% and patient was unresponsive to increased oxygen flow and repositioning of the head/neck, patients were ventilated by face‐mask/laryngeal‐mask; if endotracheal intubation was needed, an anesthetist was always immediately available. All underwent pre‐procedure TTE and where indicated pre‐op TOE and/or cardiac CT/MRI. As cases were performed using conscious sedation alone and intraoperative TOE was not utilized. All patients were asked whether they experienced an unacceptable level of pain peri‐procedure.

### 2.4 Lead extraction

Local anesthesia (1% lidocaine) was administered at the device and femoral sites in all patients. Pacemaker dependent patients received temporary right ventricular pacing via the femoral venous route. After opening the pocket, the leads were exposed, untied and if manual traction unsuccessful, a systematic approach applied using locking stylets (Liberator, Cook Medical, Bloomington, IN, USA or EZ, Philips (Spectranetics), CO, USA Spectranetic). Mechanical dilation was with polypropylene sheaths (Byrd Dilator Sheaths, Cook Medical, Bloomington, IN, USA) and when needed mechanical rotation sheaths (TightRail, Philips (Spectranetics)); the latter only became available at our center from November 2017 and laser was unavailable and not used in any cases. Snares were used via subclavian, femoral, internal jugular venous access if needed and the Needle‐Eye snare (Cook Medical) used via femoral access if needed. Swabs were used to cover the extraction site at all times to try and prevent air embolism during extraction attempt. Pacemaker dependent patients received a transcutaneous screw‐in pacemaker lead connected to an external pacemaker after removal of hardware if re‐implantation was postponed for infection. Procedure duration was defined as time of first incision to last skin suture. Complications and success rates were defined by HRS/EHRA guidelines.^9^, ^11^

### 2.5 Cost analysis

The cost for an Anaesthetist, Operating Department Practitioner (ODP) and post‐operative GA recovery bed space was obtained from our finance department. We calculated the cost savings to our hospital that occurred during the period of study for those patients who were performed using conscious‐sedation only and not requiring these additional costs. Those needing a GA and /or cardiac surgery, for whatever reason, were excluded from our cost‐saving analysis.

### 2.6 Statistical analysis

Data were analyzed using SPSS version 26.0 (IBM, Chicago, IL, USA). Continuous variables were expressed as mean ± standard deviation (SD) or median (with range) and nominal data as number (n) with percentage (%). Comparisons were made using analysis of variance (ANOVA) for continuous data and chi‐squared for categorical data.

## 3 RESULTS

### 3.1 Baseline characteristics

A total of 65 patients, with 151 leads, underwent lead extraction during this period. Of these three patients required cardiac surgery from the outset because of need for concomitant cardiac valve and/or coronary bypass surgery and were excluded. A further eight patients were also excluded as the leads were explanted without needing any extraction tools (lead dwell‐times were ≤6 months duration). This left 54 patients in total, with 130 leads in situ that underwent TLE attempt using conscious‐sedation alone (mean age: 74.6 years ± 11.8, 47 males [87%]). Baseline characteristics for the entire cohort are shown in Table 1. Bradycardia pacing indication was the leading cause for initial implantation and infections or erosions were commonest reasons for TLE. Sedation/analgesia related medications and procedural characteristics are shown in Table 2. Sedation was performed with mean ± SD drug doses as follows: fentanyl = 56.6 ± 39.7 mg, midazolam = 3.95 ± 2.44 mg, and diazepam = 4.69 ± 0.89 mg; three patients received both midazolam and diazepam. Post‐procedure none of the patients stated they had experienced unacceptable pain during or after the procedure.

**TABLE 1:** Baseline patient demographic data of the entire cohort

Demographics	Total (n = 54)
Age, mean ± SD (years)	74.6 ± 11.8
Age range (years)	36‐95
Male (n, %)	47 (87)
Height (cm) mean ± SD	173 ± 7
Weight (kg) mean ± SD mean BMI ± SD	26.4 ± 3.82
Comorbidities, n (%)	
CKD (Stage 3A or above)	14 (26)
Ischemic heart disease	26 (48)
Hypertensive heart disease	14 (26)
Diabetes mellitus	8 (15)
Cerebrovascular disease	3 (6)
Severe left ventricular systolic dsyfunction	20 (37)
Moderate left ventricular systolic dsyfunction	3 (6)
Mild left ventricular systolic dysfunction	2 (4)
Previous cardiac surgery (bypass grafting ± valve surgery)	5 (9)
Device type, n (%)	
DDDR pacemaker	26 (48)
VVIR pacemaker	2 (4)
ICD‐DR	5 (9)
ICD‐VR	3 (6)
CRT‐D	17 (31)
CRT‐P	1 (2)
Initial device indication, n (%)	
Bradycardia	29 (54)
Tachy‐Brady syndrome	2 (4)
Primary prevention	12 (22)
Secondary prevention	8 (15)
Symptomatic heart failure	1 (2)
Not documented	2 (4)
Extraction indication, n (%)	
Systemic infection	23 (43)
Device erosion	22 (41)
Lead erosion	6 (11)
Lead fracture/failure	3 (6)

**TABLE 2:** Extraction procedural data for the entire cohort

Procedure data	Total (n = 54)
Mean ± SD dwell time (years)	11.0 ± 8.8
Median (range) dwell time (years)	8.3 (0.3‐37)
Mean ± SD procedure time (min)	99.7 ± 54.0

Lead type	Right atrial	Right ventricular	ICD	Left ventricular
Total leads in situ (n = 140)	55	42	25	18
Total leads targeted for extraction (n = 130)	51	37	25	17
Complete extraction (n = 110)	43	28	25	14
Partial extraction (<4 cm retained) (n = 5)	1	2	0	2
Failed extraction (>4 cm retained (n = 15)	7	7	0	1 (Starfix lead)

Intra‐operative medication use	
Lignocaine 1% (ml) mean ± SD	33.4 ± 8.1
Midazolam (mg) mean ± SD	3.95 ± 2.44
Diazepam (mg) mean ± SD	4.69 ± 0.89
Fentanyl (µg) mean ± SD	56.6 ± 39.7

Minor complications (n = 9)	n (%)
Hypotension requiring IV fluids/atropine	2 (4)
Transient asystole or bradycardia	3 (5)
Pericardial effusion <1 cm	2 (4)
Pericardial effusion >1 cm	1 (2)
Radiographic evidence of vascular staining of SVC (no intervention needed)	1 (2)

Major complication (n = 1)	
Pericardiocentesis requiring sternotomy	1 (2)

### 3.2 Lead extraction success and complications

**FIGURE 2:**
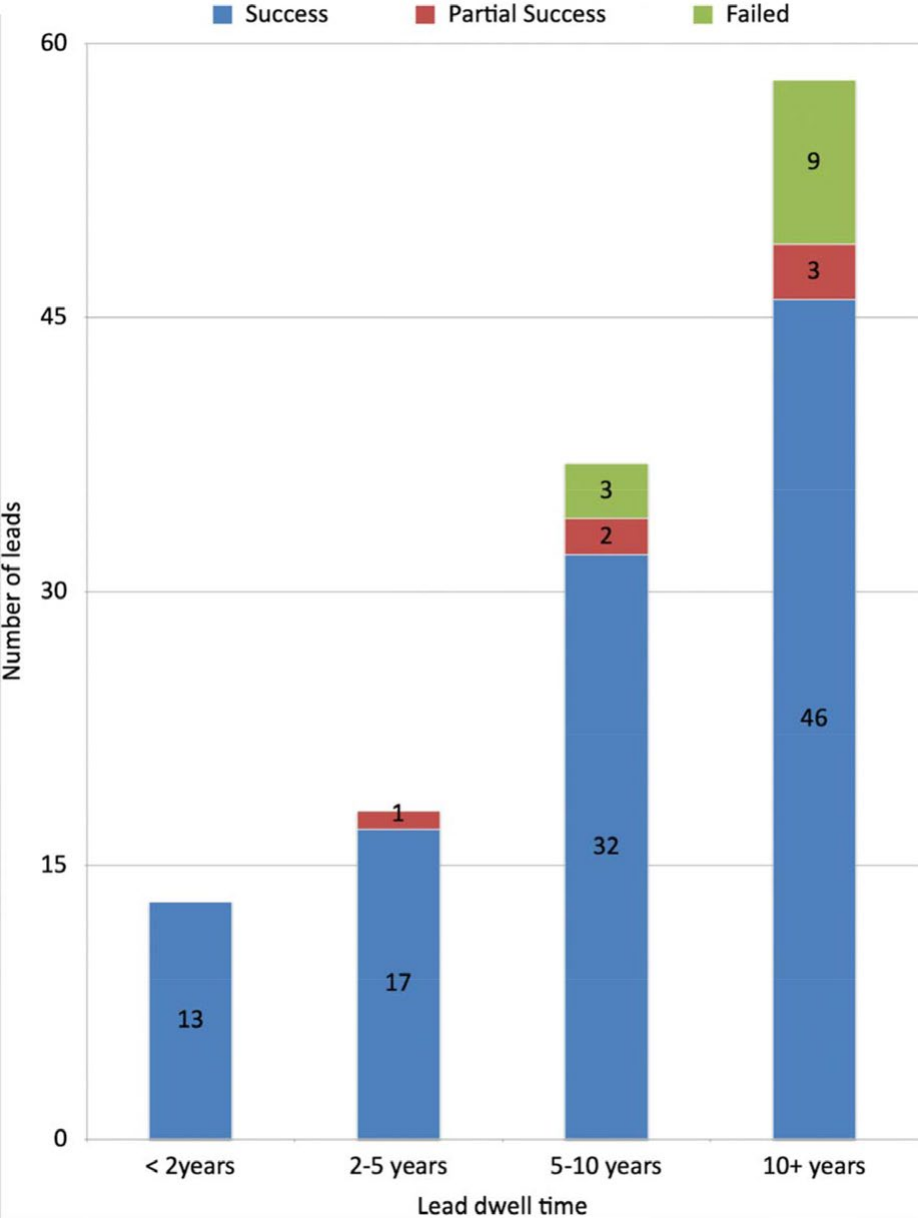
Extraction outcomes in relation to lead dwell times

Of 130 leads targeted for extraction, complete lead extraction was achieved in 110 (85%) leads, partial lead extraction in 5 (4%). Complete procedural success was achieved in 40 patients and complete clinical success in 45 patients (i.e. ≤4 cm fragment remained for 5 leads in five patients). Extraction failed (defined as either >4 cm fragment remaining/aborted procedure) for 15 leads (12%) in nine patients and none of these needed cardiac surgery. The indication for lead extraction in these nine patients was lead failure or superficial‐pocket infection only. Figure 2 shows the relationship between lead dwell times and procedural success. As expected the lead extraction failure rate was highest in those with lead dwell times >10 years.

Table 3 highlights the relationship between pre‐extraction risk and baseline patient demographic data. As expected those in the high risk group had much longer lead dwell times compared with those in the intermediate or low risk groups. Table 4 shows the procedural success and complications according to risk group. Once again, as expected, all minor and the one major complication seen were all within the high risk cohort and success rate was lower in the high risk cohort. There were no air embolic events noted in our study.

**TABLE 3:** Baseline patient demographic data according to pre‐procedural risk classification

Demographics	Low risk (n = 5)	Intermediate risk (n = 14)	High risk (n = 35)	*P*
Age mean ± SD (years)	72.0 ± 7.7	72.6 ± 10.5	75.8 ± 12.7	.613
Age range (years)	63‐81	54‐92	36‐94	
Male (n, %)	5 (100)	12 (86)	30 (86)	.663
Height (cm) mean ± SD	176 ± 4	175 ± 5.7	172 ± 7.7	.569
Weight (kg) mean ± SD Mean BMI ± SD	82.8 ± 0.6 26.9 ± 1.4	74.2 ± 9.9 24.4 ± 3.18	81.0 ± 14.3 27.0 ± 4.1	.451 .279
Comorbidities n (%)				
CKD (stage 3A or above)	1 (20)	3 (21)	11 (31)	.841
Ischemic heart disease	3 (60)	7 (50)	14 (40)	.728
Hypertensive heart disease	1 (20)	6 (53)	7 (20)	.062
Diabetes mellitus	1 (20)	3 (21)	6 (17)	.991
Cerebrovascular disease	0 (0)	1 (7)	2(6)	.777
Severe left ventricular systolic dsyfunction	2 (40)	4 (29)	14 (40)	.527
Moderate left ventricular systolic dsyfunction	0 (0)	1 (7)	3 (9)	.710
Mild left ventricular systolic dysfunction	1 (20)	0 (0)	1 (3)	.225
Previous cardiac surgery (bypass grafting ± valve surgery)	0 (0)	2 (14)	2 (6)	.256
Device type, n (%)				
DDDR pacemaker	1 (20)	8 (57)	17 (49)	.360
VVIR pacemaker	0 (0)	1 (7)	1 (3)	.695
ICD‐DR	1 (20)	1 (7)	3 (9)	.677
ICD‐VR	2 (40)	0 (0)	1 (3)	.*020*
CRT‐D	1 (20)	4 (29)	12 (34)	.783
CRT‐P	0 (0)	0 (0)	1 (3)	.758
Initial device indication, n (%)				
Bradycardia	1 (20)	9 (64)	19 (54)	.288
Tachy‐Brady syndrome	0 (0)	1 (7)	1 (3)	.672
**Primary prevention**	**2 (40)**	**2 (14)**	8 (23)	.537
Secondary prevention	2 (40)	1 (7)	5 (14)	.231
Symptomatic heart failure	0 (0)	0 (0)	1 (3)	.763
Not documented	0 (0)	1 (7)	1 (3)	.672
Extraction indication, n (%)				
Systemic infection	3 (60)	7 (50)	13 (37)	.507
Device erosion	2 (40)	5 (36)	15 (43)	.899
Lead erosion	0 (0)	1 (7)	5 (14)	.547
Lead fracture/failure	0 (0)	1 (7)	2 (6)	.834
Lead type identified, n (%)				
RA lead	3	14	34	.654
RV lead	1	12	24	.427
ICD lead	4	5	16	.232
LV lead	1	4	11	.868
LV starfix	0	0	3	.400
Dwell time (years) mean ± SD	1.33 ± 0.54	4.68 ± 2.15	15.02 ± 8.52	*<.001*

Bold indicates statistically significant value (*P* < .05).

Italics refers to statistical significance (i.e. *P* < .05).

**TABLE 4:** Procedure success and complications according to risk group

Lead extraction success, n (%) (total leads n = 130)	Low risk (n = 5)	Intermediate risk (n = 14)	High risk (n = 35)	*P*
Successful lead extractions	9 (100)	34 (97)	68 (77)	.*022*
Partial lead extractions	0 (0)	1 (3)	5 (6)	.612
Failed lead extractions	0 (0)	0 (0)	12 (14)	.*030*
Procedural success				
Clinical success	5 (100)	14 (100)	27 (77)	.078
Complete success	5 (100)	13 (93)	23 (66)	.056
Minor complications				
Hypotension requiring IV fluids/atropine	0 (0)	0 (0)	2 (6)	.053
Transient asystole or bradycardia	0 (0)	0 (0)	3 (9)	.422
Pericardial effusion <1 cm	0 (0)	0 (0)	2 (6)	.569
Pericardial effusion >1 cm	0 (0)	0 (0)	1 (3)	.758
Radiographic evidence of vascular staining of SVC (no intervention needed)	0 (0)	0 (0)	1 (3)	.758
Major complications				
Pericardiocentesis requiring sternotomy	0 (0)	0 (0)	(3)	.758

Italics refers to statistical significance (i.e. *P* < .05).

There was no association between sedation related events and procedural clinical success. One patient developed pericardial tamponade during TLE diagnosed by decreased blood pressure/reduced excursion of cardiac silhouette on fluoroscopy and confirmed by bedside TTE. This was done under conscious sedation without need for additional airway support; however a vasopressor (IV Metaraminol) was given. This same patient was transferred to the operating room (OR) after pericardiocentesis had restored hemodynamic stability but failed to control bleeding; he was intubated in cardiac theatre. At surgery he had repair of a small tear in the right atrium and right ventricular free wall; the patient had 5 pacing leads in situ (dwell time >35 years). We managed to extract all material except an old right ventricular lead tip and right atrial lead fragment using both subclavian and femoral approaches; he was pacing dependent and had a TPW inserted pre‐extraction via the right femoral vein. At cardiac surgery he had a new epicardial pacing system implanted and tunneled into the rectus sheath. He remained well post‐op and was discharged home 3 days later.

No procedure‐related deaths occurred in any of our cases. Two died within 1 month of procedure at 9 and 14 days, both unrelated to the extraction. We had two episodes of hypotension/bradycardia attributed to sedation and vagal response that responded to treatment with IV fluids/atropine. All maintained spontaneous respiration throughout the procedure. A comparison of the eight patients excluded from this analysis (three surgical GA cases and eight short dwell time/explant cases) revealed no significant differences in procedural complications compared with the conscious‐sedation group.

### 3.3 Cost analysis

The cost for an Anaesthetist, Operating Department Practitioner and post‐operative GA recovery bed space for our hospital was calculated at an average of £450 per patient. Of the 54 patients performed using conscious‐sedation, only one patient required anesthetic support with subsequent cardiac surgery. This meant 53 patients did not incur this additional cost, saving our hospital £23,850 over the study period. This cost‐saving analysis did not include the time of the cardiac surgeon or anesthetist and if taken into account would have resulted in greater cost savings being demonstrated.

## 4 DISCUSSION

Lead extraction is a complex and high‐risk intervention.^9^, ^11^ In the current study we have shown that TLE in our case series was feasible and safe; this is the first report to our knowledge using conscious sedation alone. Titrated doses of Benzodiazepines/Fentanyl were safely administered by a competent cardiology team member without needing in‐lab anesthetic support. Sedation related side‐effects were rare and managed adequately. Currently, GA is recommended for TLE with intra procedural TOE and resuscitation if needed.^9^ We found fluoroscopy, hemodynamic monitoring and bedside TTE effectively diagnosed pericardial tamponade during the procedure. Complication rates and mortality associated with TLE have been shown to be low, irrespective of whether the procedure was done in cardiac‐theatre under GA or in the EP lab with deep sedation^13^, ^14^; this is in keeping with our study. One patient in our study suffered cardiac tamponade with immediate pericardiocentesis leading to hemodynamic stabilization, but required surgical repair to control bleeding. The most serious complication, vascular tears, is associated with 50% mortality even with immediate surgical treatment; deployment of an endovascular Bridge Balloon (Philips (Spectranetics)) can help reduce this mortality.^15^ We adapted our TLE policy before availability of the Bridge Balloon; however, we have not had to use it in any cases to date.

Although performing these cases in the setting of an OR can offer immediate surgical intervention, its use adds to the scheduling complexity, cost, and resource utilization. In most centers, the EP‐lab typically has superior fluoroscopy and more ready access to percutaneous tools and support staff trained in their use. A hybrid lab with surgical capability and superior fluoroscopy, with staff trained in both lead extraction and cardiac surgical intervention, can provide the optimal balance but this type of facility is not widely available.

The ELECTRA registry^16^ indicated pericardiocentesis, followed by rescue surgery, appeared effective and safe for cardiac tamponade treatment; this supports our findings and highlights need for appropriate risk stratification pre‐intervention. The success and complication rates in our study are comparable to numerous prior studies.^13^, ^14^, ^16^, ^17^, ^18^ In our cohort, only one patient required vasopressor medication and no patient required intubation because of sedation related hypoxia. GA can facilitate airway management but is associated with risk of hypotension and possible exposure to aerosolized pathogens such as COVID‐19.^10^ In a cohort of patients with more comorbidities (85% ASA Classes III/IV) undergoing CIED surgery, the reported incidence of compromising hypoxia/hypotension under sedation by anesthetists were higher (16%/15% respectively)^19^; in our small cohort all adverse events were managed successfully.

We always limited Fentanyl to a maximum 200 µg IV as these patients can be at higher risk of sedation related complications. Well trained cath‐lab staff can manage sedation to prevent critical persistent hypotension/hypoxia, avoiding transition to GA, and be able to resolve critical situations if they appear.^13^ In the UK, use of Propofol is limited to anesthetists and therefore we were unable to use this agent. Propofol is short acting with broad use for induction of anesthesia/deep sedation; however, it’s potential to cause rapid changes in neuropsychological function, from conscious sedation to deep sedation, or even narcosis with cardiorespiratory depression/apnea should be borne in mind. However, its use in TLE has been shown to be safe and effective in high‐volume experienced centers.^13^

Risk stratification pre‐extraction is absolutely vital in helping to guide the type of anesthetic and surgical cover needed.^20^, ^21^ The aim is always to ensure TLE is timely, safe, feasible, and efficacious. Our study suggests low and intermediate‐risk procedures can safely be performed in the EP‐lab using conscious sedation with a rescue strategy, which could facilitate the provision of care in a timely manner without delay in performing the TLE procedure. High‐risk cases should be conducted under the expertise of a multidisciplinary team immediately available to allow immediate surgical intervention if needed. This may be in the EP‐lab or hybrid‐lab environment but both environments must have immediate access to GA/facilities to open the chest and perform cardiac bypass if needed. Jacheć et al^21^ have recently suggested a simple multi‐parametric algorithm to try and facilitate the prediction of potential significant complications. They proposed a SAFeTY‐TLE risk score (S = sum of lead dwell times, A = anemia, Fe = female, T = previous treatment, Y = young patients, TLE = transvenous lead extraction) with high‐risk patients (scoring >10 on the SAFeTY‐TLE scale) being treated at high‐volume centers with surgical backup. We believe an appropriate risk stratification protocol pre‐op is mandatory and can help guide resource utilization and optimize the safety and efficiency for patients needing TLE. Sidhu et al^22^ recently published a study evaluating risk stratification of patients undergoing TLE with the ELECTRa Registry Outcome Score (EROS). They risk stratified patients into low risk (EROS 1), intermediate risk (EROS 2), and high risk (EROS 3) and applied to the European Lead Extraction ConTRolled ELECTRa registry. They found patients with EROS 3 or 2 were significantly more likely to require powered sheaths/femoral approach were more likely to suffer procedure‐related major complications, including deaths, cardiac avulsion or tear and cardiovascular lesions requiring pericardiocentesis, chest tube, or surgical repair. This once again highlights the importance of appropriate risk scoring pre‐procedure.

We noted complete or partial success of TLE in 89% of leads and clinical success in 45/54 (83%) of patients. A previous published review of the efficiency and safety of TLE methods suggested higher success rates.^23^ This may be accounted for by the evolution of the technology used in our study with powered tools only becoming more widely available at our institution from 2017 onwards. The other possibility may have been that patients less likely tolerated TLE using conscious sedation compared with using GA. Our sample size was too small to be certain that the conscious sedation approach is as safe as for cases done using GA, but there is no signal of a major increase or decrease in safety. The LExlCon study^24^ indicated procedural major adverse events were higher in low volume centers than in experienced centers. Selection of conscious‐sedation or GA should be evaluated in each case and in each center with cardiac surgeon and anesthetist.

Our cost‐analysis revealed that TLE cases performed with conscious‐sedation alone can be associated with significant cost savings for healthcare providers. Our institute made a significant cost savings over the period of study and this is important in both developed and developing healthcare systems where costs are rising, especially given the current financial climate worldwide. Not all units recover post‐GA cath‐lab cases differently to those performed using conscious‐sedation. This may not avoid the cost of a recovery bed but where sedation is can be performed by specialist cath‐lab nurses; this may save on the cost of an anesthetist and ODP. In future, a policy of conscious sedation by default could offer large scale benefits in terms of quality, productivity and cost efficacy.

### 4.1 Study limitations

There are several limitations of the current study. It is a single center small retrospective study. We did not compare different anesthesia approaches and our results may not be generalizable. Our limited sample size means our results should be interpreted with caution and be considered ‘hypothesis generating.’ Although we questioned patients post procedure about their pain experience, this was limited and future studies should investigate patient pain perception and overall patient satisfaction in greater detail.

## 5 CONCLUSION

TLE using conscious sedation alone in selected patients is feasible and appears safe in centers with experienced staff. However, patient characteristics, risk predictors and preferred extraction approaches need to be carefully considered. Those deemed high‐risk must have immediate surgical/anesthetic support available if needed. Reducing the risk of aerosolized pathogen transmission, especially COVID‐19, is important in the current climate and utilizing an approach which avoids invasive ventilation can help reduce the risk of infection transmission. Additional larger randomized studies are needed to identify patient groups that might benefit from one anesthesia mode vs another.

## CONFLICT OF INTEREST

Authors declare no Conflict of Interests for this article.
